# Does the pre-conversion platform matter? A comparison of laparoscopic and robotic converted to open colectomies

**DOI:** 10.1007/s00464-024-11079-0

**Published:** 2024-07-19

**Authors:** Rachel Ma, Kristina La, Vincent Xu, Paola Solis-Pazmino, Abbas Smiley, Moshe Barnajian, Joshua Ellenhorn, Joshua Wolf, Yosef Nasseri

**Affiliations:** 1 Surgery Group Los Angeles, 8635 W 3rd St Suite 880, Los Angeles, CA 90048 USA; 2 Surgery Department, Santa Casa de Porto Alegre, Porto Alegre, RS Brazil; 3 Knowledge and Evaluation Research Unit, Mayo Clinic, Rochester, MN USA; 4 CaTaLiNA-Cancer de Tiroides en Latino America, Quito, Ecuador; 5 Department of Surgery, Westchester Medical Center, Valhalla, NY USA; 6 Department of Colon and Rectal Surgery, LifeBridge Health, Westminster, MD USA; 7 Department of Medicine, George Washington University, Washington, DC USA

**Keywords:** Colectomy, Conversion, Neoplasia

Developed in the 1980s and 2000s respectively, laparoscopic and robotic colectomies are two minimally invasive surgical approaches that offer benefits in treatment of colon cancers, including lower morbidity and shorter hospital stays, compared to their laparotomy counterparts [1–6]. Robotic approaches were initially developed to address certain limitations of laparoscopic approaches, specifically improving three dimensional vision, dexterity, and stabilization of tremors, and in colorectal surgery proved to have lower conversion rates but longer operating time and higher costs [7–12].

The conversion rates for laparoscopic colectomies and robotic colectomies are reported between 5.2 and 16.6% or 4.1–7.4%, respectively, favoring robotic surgery [13–19]. Converted minimally-invasive operations are associated with worse outcomes. For both laparoscopic converted to open (LCTO) and robotic converted to open (RCTO) colectomies, patients experience higher blood loss, longer mean hospital stays, and higher complication rates as well as higher costs [13, 15–17, 19]. Though there are many studies comparing robotic and laparoscopic colectomies, comparisons between converted minimally invasive surgeries are lacking.

This study aimed to use a large national database, the American College of Surgeons National Surgical Quality Improvement Program (NSQIP) database, to compare patient characteristics and short term outcomes following converted laparoscopic and converted robotic colectomies for colon cancers from 2012 to 2020.

## Methods and procedures

Colectomy datasets from the American College of Surgeons National Surgical Quality Improvement Program (ACS-NSQIP) database was retrospectively queried for patients who underwent elective LCTO and RCTO colectomies for colon cancers between 2012 and 2020. The database describes conversions from either platform as unplanned, but does not provide any further specifications as to size of incision or reason for conversion.

Cases involving only colon cancers were selected using International Classification of Diseases Revision Ninth/Tenth (ICD-9/10) codes. These included all codes related to malignant neoplasms of the colon: 153.0, 153.1, 153.2, 153.3, 153.4, 153.5, 153.6, 153.7, 153.8, and 153.9, or C18.0, C18.1, C18.2, C18.3, C18.4, C18.5, C18.6 and C18.7. Patients who had completely dependent functional health status, American Society of Anesthesiologists (ASA) classification IV or higher, preoperative sepsis, disseminated cancer, and those with missing demographic data points were excluded.

Pre-operative characteristics as well as intraoperative and postoperative outcomes for the two groups were identified. Preoperative characteristics included age, sex, race, body mass index (BMI), and comorbidities. Comorbidities included smoking status, diabetes mellitus, dyspnea, functional status, ventilator dependence, chronic obstructive pulmonary disease (COPD), ascites, congestive heart failure (CHF), hypertension treated with medications, renal failure, dialysis status, open wound infection, steroid use, history of weight loss in the past 6 months, preoperative blood transfusion (given within 72 h before start of operation), preoperative chemotherapy, clinical T-stage, and ASA classification. Intra and postoperative outcomes included operative time, hospital length of stay and postoperative surgical, infectious and medical complications within 30 days of operation. The complications analyzed were superficial surgical site infection (SSI), deep incisional SSI, organ space SSI, wound disruption, septic complications, septic shock, anastomotic leak, ileus, unplanned intubation, unplanned reoperation, pneumonia, pulmonary embolism, transfusion (within 72 h after start of operation), ventilator use (for more than 48 h), renal insufficiency, renal failure, urinary tract infection (UTI), stroke/cerebral vascular accident (CVA), cardiac arrest requiring CPR, myocardial infarction, and deep venous thromboembolism (DVT). Ileus was defined as abdominal distention with vomiting or necessitating insertion of a nasogastric tube without a clear transition point on imaging.

## Statistical analysis

The Software Package for Statistics and Simulation (IBM SPSS version 27, IBM Corp, Armonk, NY) was used to perform chi-square tests reported with proportions for categorical variables. Continuous variables were analyzed with ANOVA tests and reported with average value and standard deviations. Multivariate logistic regression models with backward elimination were used to evaluate association between demographics, comorbidities, operative time, and surgical approach with postoperative outcomes and reported as odds ratios (OR) and a 95% confidence interval (CI). The demographics included were age, BMI, sex, and race. The comorbidities included were functional status, tumor grade, ASA classification, preoperative chemotherapy, smoking, diabetes mellitus, hypertension treated with medications, steroid use, COPD, CHF, ventilator dependence, ascites, renal failure, open wound infection, and preoperative blood transfusion (given within 72 h before start of operation).

## Results

Our total cohort included 38,299 patients undergoing minimally invasive colectomies; 32,521 laparoscopically and 5778 robotically. Laparoscopic colectomies had a conversion rate of 9.8%, (3,200 patients), and robotic colectomies had a 4.8% (276 patients) conversion rate (*p* < 0.001). LCTO patients had a mean age of 67.5 (± 12.3) with 79.3% White, 15.1% African American, 4.9% Asian, and 0.8% American Indian or Alaskan Native. RCTO patients had a mean age of 66.0 (± 12.1) with 84.8% White, 10.5% African American, 4.0% Asian, and 0.7% American Indian or Alaskan Native. RCTO patients had lower rates of preoperative steroid medication use (1.4% vs. 3.8%, *p* = 0.046) and transfusions within 72 h before the start of operations (1.1% vs. 3.6%, *p* = 0.029). There was no significant difference between LCTO and RCTO in demographics and all other comorbidities (Table 1).

**Table 1:** Demographic and preoperative characteristics

Variables (%)	Total cases (*n* = 38,299)	Laparoscopic (*n* = 32,521)	Robotic (*n* = 5,778)	
Conversion rate	3476 (9.1%)	3200 (9.8%)	276 (4.8%)	** < 0.001**

Variables (%)	All converted (*n* = 3,476)	Laparoscopic converted to open (*n* = 3,200)	Robotic converted to open (*n* = 276)	*P*
Age, years (Mean ± SD)	67.3 ± 12.3	67.5 ± 12.3	66.0 ± 12.1	0.056
BMI, Kg/m^2^ (Mean ± SD)	29.9 ± 7.6	29.8 ± 7.6	30.4 ± 7.2	0.228
Sex				0.458
Male	1840 (52.9%)	1688 (52.8%)	152 (55.1%)	
Female	1636 (47.1%)	1512 (47.3%)	124 (44.9%)	
Race				0.172
American Indian or Alaskan Native	26 (0.7%)	24 (0.8%)	2 (0.7%)	
Asian	168 (4.8%)	157 (4.9%)	11 (4.0%)	
African American	511 (14.7%)	484 (15.1%)	29 (10.5%)	
White	2771 (79.7%)	2537 (79.3%)	234 (84.8%)	
Smoker	490 (14.1%)	450 (14.1%)	40 (14.5%)	0.844
Diabetes	818 (23.5%)	758 (23.7%)	60 (21.7%)	0.464
Dyspnea	366 (10.5%)	341 (10.7%)	9.1% (25)	0.407
Functional status^a^	92 (2.6%)	83 (2.6%)	9 (3.3%)	0.508
Ventilator dependent	2 (0.1%)	2 (0.1%)	0 (0%)	0.678
COPD	191 (5.5%)	176 (5.5%)	15 (5.4%)	0.964
Ascites	16 (0.5%)	16 (0.5%)	0 (0%)	0.239
CHF	34 (1.0%)	33 (1.0%)	1 (0.4%)	0.279
Hypertension	2126 (61.2%)	1954 (61.1%)	172 (62.3%)	0.681
Renal failure	4 (0.1%)	4 (0.1%)	0 (0%)	0.557
Dialysis	13 (0.4%)	13 (0.4%)	0 (0%)	0.289
Open wound infection	19 (0.5%)	19 (0.6%)	0 (0%)	0.199
Steroid use	125 (3.6%)	121 (3.8%)	4 (1.4%)	**0.046**
Weight loss^b^	194 (5.6%)	176 (5.5%)	18 (6.5%)	0.478
Transfusion^c^	117 (3.4%)	114 (3.6%)	3 (1.1%)	**0.029**
Pre-operative chemotherapy	68 (2.0%)	59 (1.8%)	9 (3.3%)	0.103
Clinical T-stage				0.928
T0, Tis	78 (2.2%)	71 (2.2%)	7 (2.5%)	
T1-3	2567 (73.8%)	2365 (73.9%)	202 (73.2%)	
T4, T4a-b	831 (23.9%)	764 (23.9%)	67 (24.3%)	
ASA Classification				0.593
I	41 (1.2%)	36 (1.1%)	5 (1.8%)	
II	1128 (32.5%)	1040 (32.5%)	88 (31.9%)	
III	2307 (66.4%)	2124 (66.4%)	183 (66.3%)	

Bolded *p*-values are < 0.05 and significant

^a^Partially dependent

^b^10% body weight in past 6 months

^c^Within 72 hours before start of operation

RCTO cases had significantly longer mean operative time (246.7 ± 111.9 min vs. 198.5 ± 87.6 min, *p* < 0.001) (Table 2). Postoperatively, RCTO patients had similar overall complication rates as LCTO patients (42.0% vs. 40.1%, *p* = 0.523), but had higher rates of organ space SSI (8.0% vs. 4.6%, *p* = 0.013) and septic shock (4.0% vs. 1.7%, *p* = 0.008). RCTO patients also had higher rates of unplanned reoperation within 30 days of the initial operation (8.9% vs. 5.3%, *p* = 0.002). No significant differences were found between RCTO and LCTO cases in other complications, including rates of superficial SSI (3.6% vs. 6.1%, *p* = 0.091), deep incisional SSI (0.7% vs. 0.8%, *p* = 0.875), anastomotic leak (4.3% vs. 3.4%, *p* = 0.413), or ileus (22.1% vs. 21.8%, *p* = 0.921).

**Table 2:** Intraoperative and postoperative variables

Variables (%)	All converted (*n* = 3,476)	Laparoscopic converted to open (*n* = 3,200)	Robotic converted to open (*n* = 276)	*p*
Surgical outcomes and complications				
Operative time, minutes (SD)	202.3 (90.7)	198.5 (87.6)	246.7 (111.9)	**< 0.001**
Total hospital stay, days (SD)	7.3 (8.6)	7.4 (8.6)	6.4 (8.2)	0.070
Any complication	1398 (40.2%)	1282 (40.1%)	116 (42.0%)	0.523
Superficial SSI	206 (5.9%)	196 (6.1%)	10 (3.6%)	0.091
Deep incisional SSI	28 (0.8%)	26 (0.8%)	2 (0.7%)	0.875
Organ space SSI	170 (4.9%)	148 (4.6%)	22 (8.0%)	**0.013**
Wound disruption	35 (1.0%)	31 (1.0%)	4 (1.4%)	0.443
Septic complications	108 (3.1%)	95 (3.0%)	13 (4.7%)	0.110
Septic shock	66 (1.9%)	55 (1.7%)	11 (4.0%)	**0.008**
Anastomotic leak	121 (3.5%)	109 (3.4%)	12 (4.3%)	0.413
Ileus	760 (21.9%)	699 (21.8%)	61 (22.1%)	0.921
Unplanned intubation	69 (2.0%)	60 (1.9%)	9 (3.3%)	0.113
Unplanned reoperation	178 (5.1%)	156 (4.9%)	22 (8.0%)	**0.025**
Medical complications				
Pneumonia	102 (2.9%)	92 (2.9%)	10 (3.6%)	0.480
Pulmonary embolism	36 (1.0%)	33 (1.0%)	3 (1.1%)	0.930
Transfusion^a^	463 (13.3%)	13.3% (426)	13.4% (37)	0.965
Ventilator^b^	47 (1.4%)	40 (1.3%)	7 (2.5%)	0.076
Renal insufficiency	32 (0.9%)	29 (0.9%)	3 (1.1%)	0.763
Renal failure	23 (0.7%)	20 (0.6%)	3 (1.1%)	0.364
UTI	81 (2.3%)	70 (2.2%)	11 (4.0%)	0.057
Stroke/CVA	13 (0.4%)	13 (0.4%)	0 (0%)	0.289
Cardiac arrest^c^	27 (0.8%)	24 (0.8%)	3 (1.1%)	0.541
Myocardial infarction	40 (1.2%)	37 (1.2%)	3 (1.1%)	0.918
DVT	50 (1.4%)	48 (1.5%)	2 (0.7%)	0.299

Bolded *p*-values are < 0.05 and significant

^a^Within 72 hours after start of operation

^b^For more than 48 hours

^c^Requiring CPR

We then performed multivariable regression analysis, using LCTO as the reference group. We found that RCTO patients possessed no significantly increased odds of developing any specific complications (Table 3).

**Table 3:** Multivariable logistic regression model with backward elimination evaluating the association between surgery and outcomes

Independent variables	Dependent variables							
	Any complications		Organ space surgery site infection		Septic shock		Unplanned reoperation	
	Odds ratio (95% CI)	*p*	Odds ratio (95% CI)	*p*	Odds ratio (95% CI)	*p*	Odds ratio (95% CI)	*p*
Surgical approach	Removed by Backward Elimination		1.599 (0.992–2.579)	0.054	1.899 (0.931–3.872)	0.078	1.544 (0.950–2.511)	0.080
Age	1.015 (1.009–1.022)	**< 0.001**	0.986 (0.973–0.999)	**0.040**	1.027 (1.004–1.050)	**0.021**	0.665 (0.482–0.919)	**0.013**
BMI	0.985 (0.975–0.995)	**0.002**	0.970 (0.949–0.991)	**0.005**	Removed by Backward Elimination		Removed by Backward Elimination	
Sex	0.808 (0.701–0.933)	**0.004**	Removed by Backward Elimination		Removed by Backward Elimination		Removed by Backward Elimination	
Operative time	1.003 (1.002–1.004)	**< 0.001**	1.003 (1.001–1.004)	**0.001**	1.004 (1.001–1.006)	**0.002**	1.002 (1.000–1.003)	**0.020**
Tumor grade	**0.036**		Removed by Backward Elimination		Removed by Backward Elimination		Removed by Backward Elimination	
Tis, T0	Reference Group							
T1-3	1.164 (0.715–1.894)	0.541						
T4, T4a, T4b	1.432 (0.868–2.364)	**0.160**						
ASA classification	**0.004**		0.080		Removed by Backward Elimination		Removed by Backward Elimination	
I	Reference Group		Reference Group					
II	0.827 (0.429–1.5950	0.571	0.458 (0.154–1.363)	0.161				
III	1.081 (0.561–2.082)	0.816	0.663 (0.225–1.953)	0.456				
Preoperative chemotherapy	Removed by Backward Elimination		0.223 (0.031–1.630)	0.139	Removed by Backward Elimination		Removed by Backward Elimination	
Hypertension with medication	Removed by Backward Elimination		Removed by Backward Elimination		Removed by Backward Elimination		1.367 (0.980–1.907)	0.066
Steroid medication	1.496 (1.028–2.176)	**0.035**	Removed by Backward Elimination		Removed by Backward Elimination		2.474 (1.375–4.451)	**0.003**
Severe COPD	1.593 (1.171–2.166)	**0.003**	Removed by Backward Elimination		2.710 (1.305–5.630)	**0.008**	1.063 (0.961–2.947)	0.068
Open wound infection	0.388 (0.126–1.192)	0.098	Removed by Backward Elimination		Removed by Backward Elimination		Removed by Backward Elimination	
Transfusion^a^	1.765 (1.219–2.556)	**0.001**	Removed by Backward Elimination		3.343 (1.393–8.026)	**0.007**	Removed by Backward Elimination	

Bolded *p*-values are < 0.05 and significant

^a^Within 72 hours before start of operation

## Discussion

Our study utilized a large national database to compare outcomes associated with elective laparoscopic or robotic colectomies for cancer after conversion to open. We found that conversion rates for laparoscopic (9.8%) and robotic colectomies (4.8%) fell within the ranges reported by prior literature of 5.2–16.6% or 4.1–7.4%. Consistent with prior findings, our study reveals significantly lower conversion rate in robotic colectomies [13–19]. In assessing outcomes following LCTO and RCTO, the trend favored LCTO but there were no statistically significant differences on multivariate analysis.

On univariate analysis, RCTO cases had significantly longer mean operating times, around 25% or 48.2 min longer, than LCTO cases. This result was supported in most prior studies comparing robotic and laparoscopic surgeries [2, 20, 21]. RCTO was not associated with a higher odds of overall complications or any specific complication. NSQIP included a relatively small number of RCTO cases compared to LCTO cases. This reduced the power of our statistics. Future studies with a larger number of patients in the two comparative groups may indeed show differences in outcomes.

Feng et al. reported on a recent multicenter randomized controlled trial across 11 institutions comparing postoperative outcomes for patients undergoing laparoscopic versus robotic resections for rectal cancers. They found that robotic surgery was associated with lower rates of overall postoperative complications, faster gastrointestinal recovery, and shorter postoperative hospital stays [20]. Similar to our findings, this study reported a significantly lower rate of conversion in robotic surgery. This multicenter study and others have shown worse postoperative outcomes following laparoscopic compared to robotic surgery. Our study has shown that this discrepancy in post operative outcomes does not hold true for cases that have been converted.

A 2015 meta-analysis by Chang et al. reported compared outcomes following robotic and laparoscopic colectomies in 125,989 cases. Similar to our findings, they found longer operating times and lower conversion rates in robotic colectomies. Similar to Feng et al., Chang et al. also found a significantly lower complication rate and faster recovery of gastrointestinal function following robotic surgeries [2]. Chang et al. hypothesized that prior abdominal surgery is a factor for conversion. Prior abdominal surgery can act as an exclusion factor in future studies to ensure that it does not confound conversion rates or other postoperative outcomes. Separately, Chang et al., as opposed to our study, did not exclude emergent surgeries or patients with severe comorbidities, which may have confounded their results.

In 2022, Solaini et al. performed a meta-analysis analyzing postoperative outcomes following laparoscopic or robotic left colectomies for cancers. In the following year, Zheng et al. performed the same analysis but for right colectomies. Both studies found lower conversion rates and longer operating times in robotic as compared to laparoscopic colectomies [11, 22]. Both Zheng et al. and Solaini et al. hypothesize that the lower conversion rates in robotic surgeries are due to the freedom of movement, visualization, and stabilization of platform’s machinery. These factors better equip the surgeon to operate in small spaces and remove more serious lesions, which may otherwise have prompted conversion. The increased maneuverability in robotic surgeries may lead to a higher threshold for conversion as compared to laparoscopic surgeries. While Zheng et al. showed a similar rate of postoperative complications in robotic versus laparoscopic right colectomies, Solaini et al. reported a lower rate of complications following robotic left colectomies [11, 22]. The side of colon resection may act as a confounder in the outcomes of converted MIS in our study as we pooled all side colectomies together. As NSQIP reports a larger number of RCTOs, we will be able in the future to run separate analyses for LCTO and RCTO for right colectomies and left colectomies.

The main strength of this study is that it is the only study that compares outcomes following converted cases from robotic and laparoscopic surgeries. Though studies comparing robotic and laparoscopic surgeries are prevalent, as well as studies on consequences of conversions, there are no head to head studies comparing outcomes following conversion from robotic and laparoscopic operations [7–12]. Another strength is the use of a large administrative database that is nationally representative and prospectively collected in a consistent manner by dedicated clinical reviewers. NSQIP draws from more than 700 medical centers, increasing the generalizability of the results.

Several limitations of this study should be addressed. As mentioned, the small number of patients in the robotic group limits the power of our results. Furthermore, due to the small number of patients, we grouped left, right, and transverse colectomies together, even though each of these have their respective operational challenges and patient risks. Additionally, large databases, such as NSQIP, inherently contain coding inconsistencies that may produce type II errors and can produce statistically significant results that may not be considered clinically significant, and selection bias is inherent to retrospective studies like ours. Furthermore, trends observed in this study may not be representative of non-NSQIP participating hospitals. Surgeon selection bias could not be assessed as NSQIP does not report on why a certain surgical approach was selected for a particular patient. Furthermore, reported outcomes were limited to 30 days post-operation, and clinically significant long-term outcomes could not be assessed. Other important parameters are not currently reported in the NSQIP database, including granular data on prior surgeries, surgeons’ level of experience, medical center care protocols, and more, which all could confound the results. Another limitation of our paper is the fact that the learning curve of the surgeon, especially with the newer robotic platforms, is likely a significant confounding factor, but it is not captured by the NSQIP data.

In light of the lower conversion rates of robotic colectomies, one might conclude that the threshold for conversion is higher and would therefore lead to higher morbidity as compared to laparoscopic colectomies. However, we found that robotic colectomies not only have a lower rate of conversion, but also that conversion is associated with similar postoperative outcomes as compared to converted laparoscopic colectomies. The results of this study may better inform surgeons and patients preparing for minimally invasive colectomies.
